# The Moderating Effects of Gender and Study Discipline in the Relationship between University Students’ Acceptance and Use of ChatGPT

**DOI:** 10.3390/ejihpe14070132

**Published:** 2024-07-08

**Authors:** Ibrahim A. Elshaer, Ahmed M. Hasanein, Abu Elnasr E. Sobaih

**Affiliations:** 1 Management Department, College of Business Administration, King Faisal University, Al-Ahsa 31982, Saudi Arabia; ielshaer@kfu.edu.sa (I.A.E.); aabdelrazek@kfu.edu.sa (A.M.H.); 2 Faculty of Tourism and Hotel Management, Suez Canal University, Ismailia 41522, Egypt; 3 Faculty of Tourism and Hotel Management, Helwan University, Cairo 12612, Egypt

**Keywords:** AI learning tools, ChatGPT, study discipline, UTAUT, technology usage, higher education

## Abstract

The intensive adoption of ChatGPT by university students for learning has encouraged many scholars to test the variables that impact on their use of such AI in their learning. This study adds to the growing body of studies, especially in relation to the moderating role of students’ gender and their study discipline in their acceptance and usage of ChatGPT in their learning process. This study expanded the Unified Theory of Acceptance and Use of Technology (UTAUT) by integrating gender as well as study disciplines as moderators. The study collected responses from students in Saudi universities with different study disciplines and of different genders. The results of a structural model using Smart PLS showed a significant moderating effect of gender on the relationship between performance expectancy and ChatGPT usage. The results confirmed that the impact of performance expectancy in fostering ChatGPT usage was stronger in male than in female students. Moreover, social influence was shown to significantly affect males more than females in relation to ChatGPT usage. In addition, the findings showed that study discipline significantly moderates the link between social influence and ChatGPT usage. In the same vein, social influence significantly influences ChatGPT use in social sciences more than in applied sciences. Hence, the various implications of the study were discussed.

## 1. Introduction

Artificial intelligence (AI) has recently become a research theme in many disciplines, including risk management, accounting, finance, management, and education [1,2,3]. Studies that have concentrated on the impact of AI on education have discussed many positive facets of using AI in higher education. These facets include increasing the quality of methods and instruments used by university lecturers and professors [4]. Moreover, other studies have concentrated on student use of AI, especially ChatGPT, in their essay and/or thesis writing, their activities in lectures and tutorials, and as an analytical tool in many disciplines [4]. Further, Hasanein and Sobaih [5] explained that AI can create and help to adapt new innovative teaching methodologies and can change how knowledge is transmitted and absorbed drastically. Having said this, there have been several attempts to study and understand the role of AI in educational sector; however, these early endeavors represent early studies and still there are many issues that have not yet been addressed in the literature [4,5,6]. One of these gaps is the current study’s focus on gender impact on the acceptance and usage of AI platforms in educational institutions. To find out what people’s opinions are regarding the factors that motivate the usage of AI platforms, e.g., ChatGPT in education, Hasanein and Sobaih [5] conducted a qualitative study with a variety of educational stakeholders, including students. Twelve factors were found to impact students’ perception of using chatbots for educational functions. These factors included issues like usability, help with homework, prompt response, language proofreading, troubleshooting, studying for exams, analysis of data and research assistance, self-learning, concept definition, learning materials, and evaluation tools. Strzelecki [6], adopting the Unified Theory of Adoption and Usage of Technology (UTAUT), evaluated the variables affecting the adoption and usage of ChatGPT amongst higher education Polish students. However, Strzelecki’s [6] study also highlighted the importance of future research in evaluating variations in ChatGPT usage among students, taking into account factors like gender and discipline. This is essentially the field of investigation that the current research wants to address.

The research rationale of the study is centered on the nuanced exploration of gender and disciplinary variations in the utilization of ChatGPT within higher education. Several studies (e.g., [7,8,9,10]) have acknowledged the potential distinctions between male and female students and across diverse academic disciplines in the adoption of and engagement with e-learning, i.e., AI. However, there exists a need for a more comprehensive understanding of these differences regarding ChatGPT in particular. Moreover, studies (i.e., [6,11,12,13]) have suggested that ChatGPT has played a pivotal role in diminishing the technological gap between males and females. This study further explores the unique ways in which students, grouped by gender and academic field, engage with and exploit ChatGPT in the higher education setting. By examining these differences, this study attempts to give insights that might drive specific ways of enhancing the adoption of ChatGPT in educational contexts, providing acceptance, efficiency, and relevance among varying student and disciplinary groups. In light of the growing interest in employing ChatGPT for academic functions, the current study investigates whether Saudi Arabian students in higher education institutions accept it with open arms. Through an investigation of the factors impacting the perception of students and their utilization of AI platforms, i.e., ChatGPT, in the classroom, scholars and educational establishments can acquire a more profound comprehension of these occurrences and enhance their ability to regulate the ways in which students use artificial intelligence technologies into their lectures. In order to examine students’ perceptions towards using AI platforms for educational purposes, the conceptual framework of UTAUT has been adopted for use on school of business students in Saudi Arabia. In such culturally segregated nations, e.g., Saudi Arabia, which prohibit gender interaction between men and women, the current research also seeks to explore the role of gender in the acceptance and usage of AI tools, i.e., ChatGPT, for educational purposes. Moreover, the current research aims to explore the role of study disciplines (e.g., business, education, humanities, and social sciences) affecting students’ acceptance and usage of AI platforms.

## 2. Literature Review

### 2.1. Theoretical Framework

The conceptual framework of UTAUT has been adopted due to its comprehensive framework of acceptance and usage of AI platforms in different contexts, especially for educational purposes [14,15,16]. The core aspects of the UTAUT framework, which mainly affect users’ behavioral intentions, are performance expectancy (PE), effort expectancy (EE), social influence (SI), and facilitating conditions (FC) [15,17]. Performance expectancy (PE) tends to explore students’ perception of using AI to enhance their educational proficiency [14]. As mentioned by several studies (e.g., [18,19,20,21,22,23]), students mainly rely on using AI in their hard educational activities because they believe it may assist them in critical activities and enhance their performance. In terms of EE, this indicates students’ perceptions of how simple or difficult it is to use the technology. Students’ perception of AI tools like ChatGPT being intuitive, user-friendly, and effortlessly incorporated into regular educational activities improves their behavioral intention (BI) [19]. Elements like user interface design, general user experience, and simplicity of engagement all have an impact on students’ impressions [6,23,24,25]. Considering ChatGPT to involve minimum effort enhances the possibility that students would incorporate it into their usual learning routines [5,24,26].

SI investigates how educators and fellow students influence students’ attitudes toward ChatGPT [16,24]. This effect may be shown in how students perceive others’ attitudes, beliefs, and actions about the use of ChatGPT in the classroom [15,17]. The findings of empirical studies (e.g., [27,28,29]) have shown that when classmates utilize ChatGPT for learning purposes, it has the potential to enhance the performance of other students’ BI. FC evaluates the availability of resources required for efficient use [16]. The FC element includes technical mobility, access to appropriate instruction and tools, and broad incentive programs for integration [6,27,30]. Students’ BI refers to their intention to utilize AI learning platforms, e.g., ChatGPT. This is highly impacted by their view that the learning environment provides the resources and help required for its successful implementation [5,31]. A study conducted by Al-Emran et al. [23] deepened the emphasis of FC over technological facilities and improved students’ ability to interact with technology by highlighting the necessity for a favorable environment that supports students’ navigation in the effective usage of ChatGPT. Thus, we are prompted to develop the following hypotheses:

**Hypothesis** **1–4.** *PE, EE, SI, and FC considerably and positively affect students’ BI toward usage of ChatGPT.*

Both PE and EE explore the students’ interaction e.g., perceived value, efficiency, simplicity, and appropriateness towards their actual usage of integrated platforms of AI, focusing on ChatGPT (ex., [25,26,27]). In addition, enhancing students’ contributions, completing tasks, and academic performance are considered the most crucial facets of PE in using AI in higher education [21,27]. EE incorporates several beneficial aspects, such as flexibility, ease of access, and ability to complete routine tasks, which positively influence students’ intention to use AI platforms [3]. In terms of SI, it is simply related to the previous experiences of colleagues and recommendations towards intention to use AI tools for educational purposes (ex., [24,25,26,27,28]). The choice of students to actively integrate ChatGPT into their regular educational activities is also impacted by the encouragement and favorable experiences that colleagues provide [5,23,27]. The significant influence of FC on adoption and actual usage has been highlighted by earlier studies (ex., [32,33]) evaluating ChatGPT’s adaptability in education. Menon and Shilpa’s research [27] revealed that FC, comprising a mobile device, steady connection to the internet, and technical help and assistance, are essential elements dramatically determining ChatGPT adoption. Therefore, we hypothesize that:

**Hypothesis** **5–8.** *PE, EE, SI, and FC considerably and positively affect students’ usage of ChatGPT.*

One of the most pivotal roles in comprehending the beneficial characteristics of using AI tools is to explore the link between students’ behavioral intention to use AI platforms in educational themes [34]. The behavioral intentions of users reflect their alignment toward accepting and using a new innovative technology. e.g., AI platforms [16]. The positive behavioral intention towards students’ usage of AI applications for educational purposes is considered a crucial motive for their actual usage during their daily educational tasks (ex., [35,36]). Recent studies have argued that a strong positive correlation exists between students’ BI and the actual use of AI in an educational context due to the critical help needed to complete their educational activities [17,32]. Thus, drawing upon these insights, we are driven to construct this hypothesis:

**Hypothesis** **9.** *Students’ BI considerably and positively affects students’ usage of ChatGPT.*

### 2.2. The Role of Gender and Discipline in Students’ Perceptions, Acceptance, and Use of ChatGPT

Saudi Arabia adheres to socio-religious beliefs that incorporate gender segregation, a practice evident in higher education institutions [7]. This segregation extends to virtual environments due to the sensitivity and significance of avoiding gender mixing in Saudi society [8,9]. According to Alhazmi and Nyland [10], female students are completely isolated from their male peers during their academic career, even while they are interacting with tutors or lecturers. E-learning improves females’ access to higher education, according to studies on cultures with gender-based segregation, such as Saudi Arabia [11,12]. Because of their e-learning experiences, female students may therefore be more likely than male students to use AI chatbots, such as ChatGPT [10]. Alotabibi and Alshehri [13] argued that there are variations in the adoption and utilization of ChatGPT by students across various fields in higher education. According to Alotaibi and Alshehri’s [13] study, technical fields like computer science and engineering would be more receptive to ChatGPT, seeing it as a useful means for analysis of data and tackling issues. Jabeur et al. [37] have observed that while students studying the arts and social sciences may use it for language and text analysis tasks, business students may emphasize its usage in decision-making processes. The numerous uses of ChatGPT, including its ability to adapt to the unique requirements of integrated areas, may be highlighted in interdisciplinary courses. Alotaibi and Alshehri [13] also mentioned that these variations demonstrate the importance of considering disciplinary situations while using ChatGPT for educational purposes. Strzelecki and ElArabawy [38] conducted a recent study in which they investigated the role of gender and level of education on Egyptian and Polish undergraduate students’ adoption and use of generative AI platforms (e.g., ChatGPT). The main conclusions stated that, in both nations, the influence of students’ acceptance (i.e., effort anticipation, social influence, and conducive settings) on behavioral intention and usage of AI in educational contexts was moderated by gender and study field. Building upon these discussions, we postulate these hypotheses:

**Hypothesis** **10–13.** *Gender plays a moderating role in the effects of PE, EE, SI, and FC on students’ usage of ChatGPT.*

**Hypothesis** **14–17.** *Discipline plays a moderating role in the effect of PE, EE, SI, and FC on students’ usage of ChatGPT.*

**Figure 1:**
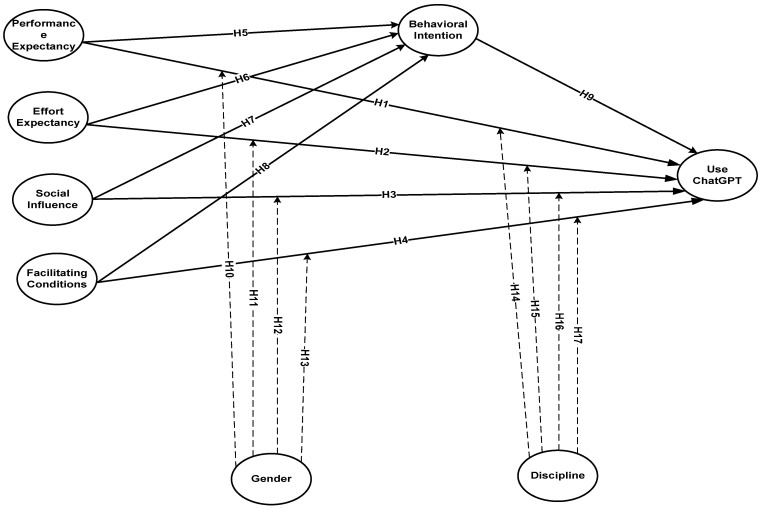
The study model.

A summary of all research hypotheses is presented in the study model (Figure 1). 

## 3. Methods

### 3.1. Participants

The population studied by this research comprised all undergraduate students in Saudi universities who use ChatGPT for learning purposes. The sample included students at public universities in different Saudi regions, including eastern, western, northern, and southern. Therefore, we had good participation of students from seven universities. There were about 70 participants from each university. We were able to collect 500 completed questionnaires that could be used for data analysis. Data collection with 500 participants is considered sufficient for conducting PLS-SEM models and meets all the suggested requirements. Among the requirements, the most important preconditions to use in the process of defining a PLS-SEM analytical model included the determination of the minimum sample size. The minimum sample size of respondents can be calculated by the “ten-times rule” method proposed by Hair et al. [39], in which the sample size must be ten times more than the number of the constructs’ variables in the model. Specifically, there are six latent factors, with twenty-one formative items and two moderators in the presented model. Therefore, applying the rule ten times, the minimum sample size required would be 230. The target population included 500 participants, which exceeded the minimum number. In addition, the sample of 500 was larger than the recommended sample size for SEM, which ranges between 100 and 150 cases if we desire to obtain reliable results [39], and the minimum sample size of 384 if the population was greater than 100,000, as suggested by Krejcie and Morgan (40). Out of these collected forms, there was more participation from males (306) than females (194) (see Table 1). In terms of students’ age groups, the majority of students (52.2%) were in the category from 20 to 25 years of age. Additionally, students’ participation in the social sciences and humanities was slightly higher. This included 262 participants from the Colleges of Art, Education, and Management, compared to 238 natural, applied, and basic sciences students (i.e., Colleges of Science, Medicine, Pharmacy and Engineering).

### 3.2. Instrument

This study used a pre-examined questionnaire to gather data from students. The questionnaire form had four main parts. The first page of the questionnaire included some information about the topic, the purpose of the study, and a request for the participants’ consent to contribute to the study. Part one included questions related to participant demographics, e.g., gender and study discipline. Part two had questions about PE, EE, SI, and FC. The questions in this part adopted a five-point Likert scale. All items in this part had been tested in previous studies [6,38] and were originally developed from UTAUT framework [15,16]. Part three also used pre-tested five-point Likert scales about the intention and use of ChatGPT for learning. Items were taken from previous studies [6,38]. There was also a space for participants to add further comments in part four. The questionnaire form was checked for content and face validity by 10 professors, and minor edits were made to the format and contents. The research items used in this study are presented in the Appendix A. Appendix A shows the complete scale of the study variables.

### 3.3. Procedures

The research team self-administered the questionnaire forms to students at the seven public universities. The procedures of data collection started with approval of the questionnaire and procedures of data collection by the ethical committee at King Faisal University. Once the instrument and procedures were approved, we contacted several universities to address their students for participation in our study. Contacts were made with the Deans of Students Affairs at the universities. The researchers then visited universities and questionnaire forms were distributed to students individually at campuses. The purpose of the study was explained to each participant, and they were all assured that the data collection was for study purposes. Their participation was anonymous; thus, no personal information was collected. Each participant gave his/her consent for participation in the study. Students were informed that their responses would not be identified nor shared with their tutors nor their university administration, in order to avoid any power bias. 

### 3.4. Data Analysis 

The PLS-SEM approach was adopted as the main data analysis process. Hair et al. [39] argued that PLS-SEM represents another method for dealing with traditional covariance-based SEM [CB-SEM]. PLS-SEM and has become more reliable, especially in prediction and exploration-focused research [40,41]. PLS-SEM works well with both small and large samples because it is not dependent on the assumption that the sampling has a normal distribution [39]. SmartPLS 4 software was utilized to analyze the PLS [41]. The bootstrapping process, using reflective mode and n = 5000 resamples, was conducted to estimate the model [42]. As discussed by [41], we adopted two steps in PLS-SEM processing: (1) assessing the outer model or measurement model for construct validity and reliability, and (2) assessing the inner or structural model (for hypotheses testing). Additionally, Harman’s one-factor test was undertaken to deal with common method variance (CMV) as proposed by Podsakoff et al. [43]. All the questions were submitted to the exploratory factor analysis (EFA), and the first component contributed 42% of the total variation. Consequently, the abovementioned result indicates that the CMV was not a major problem in this research. Furthermore, all the VIF values were below 0.5, showing that there was no significant multicollinearity (see Table 2).

## 4. Results

The assessment of the outer model, specifically the measurement model, involved scrutinizing the psychometric characteristics of diverse scales, applying criteria such as Cronbach’s α, composite reliabilities (CR), and average variance extracted (AVE]. All items within the scales exhibited standardized loadings equal to or exceeding 0.7, indicating commendable convergent validity. The values for both Cronbach’s α and CR exceeded the established minimum threshold of 0.7, affirming the internal consistency of both items and constructs (refer to Table 2). Furthermore, the AVE values for all constructs surpassed the suggested threshold of 0.5, as outlined by Fornell & Larcker [44]. Hence, the convergent validity was approved as satisfactory, as all AVEs were 0.5 or higher.

Adhering to the methodology proposed by Fornell and Larcker [44], we verified discriminant validity by confirming that the square root of the AVE for each construct (values highlighted in bold in Table 3) surpassed the correlations between that specific construct and all other constructs (refer to Table 2). Furthermore, to enhance the assessment of discriminant validity, we employed the heterotrait-monotrait (HTMT) ratio of correlations, recognized as a more robust method compared to Fornell and Larcker’s [44]. The potential for concerns regarding discriminant validity arises when HTMT values (enclosed in brackets in Table 3) surpass 0.9. As depicted in Table 2, all ratios fell below the designated threshold of 0.9, thereby confirming the discriminant validity of the measurements for the constructs.

**Figure 2:**
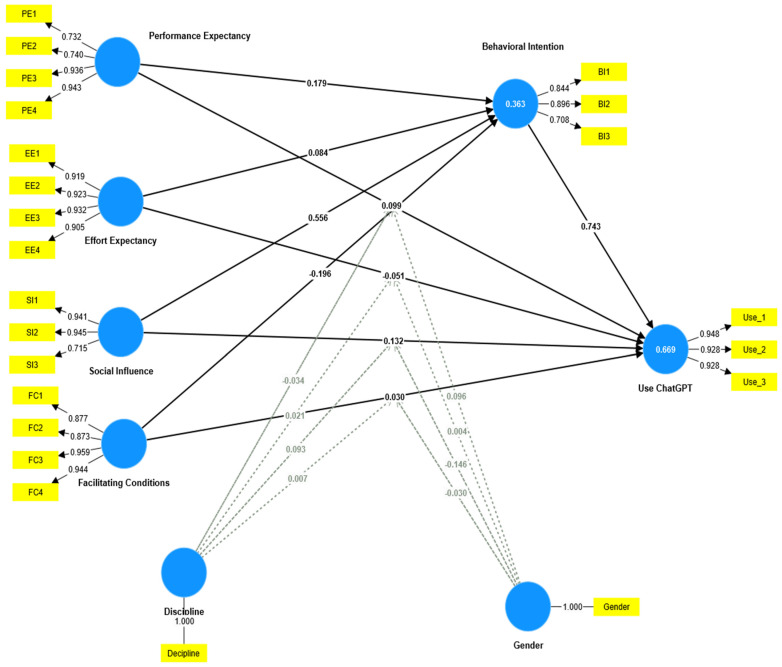
The examined relationships.

The bootstrapped R2 values, illustrated in Figure 2, revealed that the combined impact of UTAUT dimensions (performance expectancy, effort expectancy, social influence, and facilitating conditions) accounted for 36.3% of the variance in the intention to use ChatGPT. Additionally, when considering both the UTAUT dimensions and the intention to use ChatGPT, the collective explanatory power increased to 66.9% for the actual usage of ChatGPT.

Examining the bootstrapped path coefficients (Table 4), it was observed that student behavioral intention to use ChatGPT was significantly and positively impacted by performance expectancy (β = 0.179, t = 5.083, *p* < 0.001), effort expectancy (β = 0.084, t = 2.085, *p* < 0.05), and social influence (β = 0.556, t = 9.570, *p* < 0.001), supporting H1, H2, and H3. However, the results revealed that facilitating conditions negatively and significantly impacted student behavior in relation to using ChatGPT (β = −0.196, t = 4.758, *p* < 0.001), rejecting H4. Furthermore, the SEM-PLS findings provided evidence that the student’s actual usage of ChatGPT was positively and significantly impacted by performance expectancy (β = 0.099, t = 2.213, *p* < 0.05) and social influence (β = 0.132, t = 2.390, *p* < 0.05), supporting H5 and H7. However, effort expectancy (β = −0.051, t = 1.073, *p* = 0.283) and facilitating conditions (β = 0.030, t = 534, *p* ≤ 0.593) failed to influence the students’ actual usage of ChatGPT positively and significantly, rejecting H6 and H8. Finally, the behavioral intention to use ChatGPT was found to have a high positive and significant impact on the students’ actual usage of ChatGPT (β = 0.743, t = 20.283, *p* < 0.001), supporting H9.

To test the moderating impacts of gender and study discipline on the variables within the tested model, the Smart PLS-SEM v4 program was used to split the data and categorize responses according to gender (males coded as one, females coded as zero) and study discipline (social sciences coded as one, natural sciences coded as zero). As illustrated in Table 4, gender (male/female) as a moderator failed to demonstrate any significant differences in the impact of effort expectancy on ChatGPT use (β = 0.004, t = 0.076, *p* = 0.940), rejecting H11. Similarly, gender as a moderator failed to demonstrate any significant differences in the impact of facilitating conditions on ChatGPT use (β = −0.030, t = 0.538, *p* = 0.591), rejecting H13. Furthermore, discipline (social science/natural science) as a moderator failed to demonstrate any significant differences in the impact of performance expectancy on ChatGPT use (β = −0.034, t = 0.538, *p* = 0.591), effort expectancy on ChatGPT use (β = 0.021, t = 0.402, *p* = 0.688), and to make any significant differences in the impact of facilitating conditions on ChatGPT use (β = 0.007, t = 0.107, *p* = 0.915) rejecting H14, H15, and H17.

**Figure 3:**
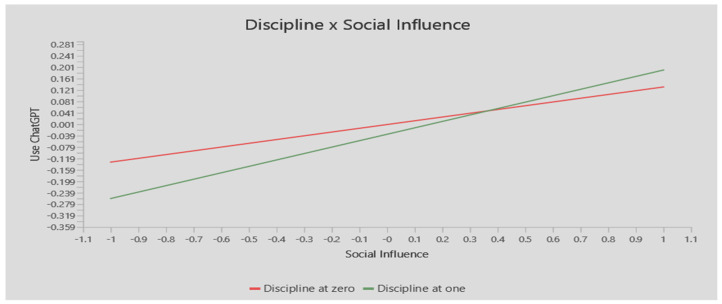

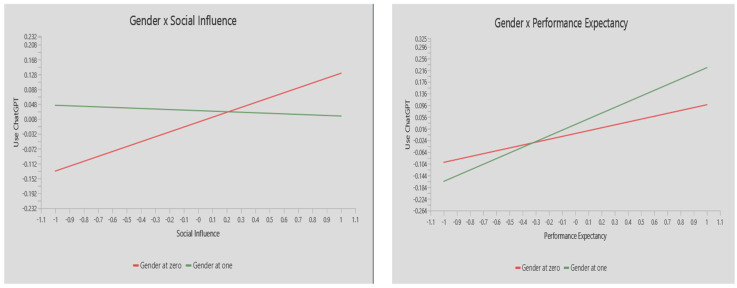
The moderation effects. Gender: males coded as one, females coded as zero. Discipline: social sciences coded as one, natural sciences coded as zero.

On the other hand, as shown in Figure 3, the results revealed a significant moderating role of gender in the link between performance expectancy and use of ChatGPT. The plot shows a steeper and positive gradient for males compared to females. Thus, this shows that the impact of performance expectancy in fostering ChatGPT usage is stronger in males as compared to females, supporting H10. Moreover, the findings demonstrated that gender significantly modified the association between behavioral intention and social impact. In comparison to the male plot, the female plot displays a steeper and more positive gradient. This supports H12 by demonstrating that social influence has a greater effect on females than on males in promoting ChatGPT usage. The research discipline significantly moderated the link between social influence and ChatGPT use, as the data showed. In comparison to the natural sciences, the social science subject was represented by a plot with a steeper and more positive gradient. This demonstrates that social influence has a greater influence on ChatGPT use in social sciences than in natural sciences, which supports H16.

## 5. Discussions

This research adopted the UTAUT framework to test the variables that influence students’ use of ChatGPT in learning. This study focused on how gender and academic discipline moderate the relationship between students’ approval and use of ChatGPT for learning. The findings verified that PE, EE, and SI greatly and favorably influenced the desire of students to adopt ChatGPT for learning. These data support H1, H2, and H3, respectively. These results align with the hypotheses proposed by the UTAUT structure [15,16], which verified a statistically significant impact of the three factors (PE, EE, and SI) on the behavioral intention to adopt ChatGPT for e-learning. The data confirmed that since students found ChatGPT to be an acceptable instrument for their academic pursuits, it encourages them to attain their academic objectives, boosts their productivity, and elevates their academic performance. The results of this study validate the claims stated by earlier research [6,21,22,23,38], showing that PE positively affects the behavioral intention to adopt ChatGPT for learning. The current study also confirmed that students found that ChatGPT does not require much effort to learn how to use, hence showing positive intention to adopt it for learning. These findings are supported by previous studies (e.g., [6,25,26,27]) which have shown that when users find that an AI tool is user-friendly and easy to comprehend, they express positive intention to adopt the tool, such as with ChatGPT for learning. Furthermore, this study has supported previous studies [15,17] in the assertion that SI shaped by colleagues and tutors positively impacts behavioral intentions to embrace ChatGPT for learning. 

However, the findings showed that FC negatively and significantly impacts on students’ behaviour in terms of using ChatGPT, rejecting H4. This finding does not support the UTAUT theory [15,16], nor previous research, which has confirmed that FC significantly influences students’ behaviour towards the adoption of ChatGPT [23,31]. Previous studies have confirmed that FC variables, such as technology accessibility, providing of essential facilities, and support, positively drive students’ intentions to adopt ChatGPT for learning. The negative impact found in this study was because the quality of support and resources surrounding the use of AI tools, including ChatGPT, in learning did not meet students’ expectations. Since students did not receive quality support from their institutions or other external sources as expected, they expressed a negative intention to adopt this AI instrument for learning. H5 and H7 were supported by the SEM-PLS findings, which demonstrated that PE and SI had a direct, favorable, and significant impact on students’ usage of ChatGPT. These findings supported the UTAUT framework [15,16] and previous studies (e.g., [6,27]), in which PE and SI stand out as key factors influencing students’ usage of ChatGPT for educational purposes. However, the findings showed that EE and FC failed to positively and significantly influence the usage of ChatGPT, rejecting H6 and H8. This contradicts the UTAUT framework [15,16] and previous studies [23,26,27,31], which have confirmed the significant positive impact of EE and FC on the usage of ChatGPT for learning. Students did not have the quality support and resources from their institutions as expected; while they found that ChatGPT is user-friendly, this was not enough to ensure the usage of ChatGPT for learning. Furthermore, the behavioral intention to use ChatGPT was found to have a highly positive and significant impact on student usage of ChatGPT for learning, supporting H9. This is consistent with UTAUT theory [15,16] and previous studies [17,32]. This means that when students decided to use ChatGPT for learning, they continued their usage and found that it positively impacted their performance [45]. However, it is important that students recognize the ethical concerns related to the use of AI tools in their learning in order to maintain a sustainable impact on their performance [45,46]. 

The results confirmed a significant moderating effect of gender in the link between PE and using ChatGPT for learning. The findings confirmed that PE fosters ChatGPT usage more strongly in males compared to females, supporting H10. Additionally, the findings revealed a significant moderating role of gender on the relationship between SI and use of ChatGPT. The findings showed that the impact of SI in fostering the use of ChatGPT was stronger in females as compared to males, supporting H12. However, there was no moderating role of gender in the relationship between EE, FC, and the use of ChatGPT for learning. Previous studies in the Saudi context confirmed significant variance between males and females in their acceptance and adoption of e-learning [7,10]. These studies found that female students exhibited greater acceptance and use of e-learning because they have less direct contact with their tutors and often use learning management systems for communication. Similarly, in this research, it was revealed that female students found ChatGPT to be a more valuable tool that enhances their productivity and elevates their performance moreso than their male colleagues, and hence they are more likely to use it than male students. Female students are more likely to be affected by their peers, and hence they are more likely to use ChatGPT for learning than their male colleagues.

The results confirmed a significant moderating role of study discipline on the link between SI and use of ChatGPT. This study showed that the impact of SI in fostering ChatGPT usage was stronger in the social sciences discipline as compared to the natural sciences discipline, supporting H16. Social sciences students were found to be more affected by their peers in relation to the use of ChatGPT for learning than students in the natural sciences. There were no differences between students in different sciences regarding the effects of PE, EE, and FC on the use of ChatGPT for learning. 

This study has significant implications for scholars and higher education policy makers. It revealed the variables that impact on both students’ intention and usage of AI instruments, e.g., ChatGPT. The study confirmed that FC failed to directly and indirectly affect ChatGPT use for learning. In fact, it was found that it had a significant negative impact on behavioral intention. These findings demonstrate the lack of quality support given to students in relation to the use of ChatGPT in learning. Students are using ChatGPT for learning, but institution leaders are not encouraged to integrate AI tools in learning [5], leading to a lack of quality support. The negative impact of this on behavioral intention and its insignificant impact on usage requires developing a policy and procedures for integrating AI tools in learning. A support unit should be established to manage this process. 

The moderating effect of gender in the association between PE, SI, and ChatGPT use for learning reflects a shift in students’ use of AI tools for learning in Saudi Arabia. This is because earlier research [7,10] confirmed that female students use e-learning more than their male counterparts. This study confirms that male students found AI tools, i.e., ChatGPT, more valuable for their academic pursuits than their female counterparts. They are more likely to be affected by their peers when using ChatGPT. In addition, students in social sciences in Saudi Arabia are more likely to be affected by their colleagues than those in natural sciences regarding their use of ChatGPT for learning. This provides the opportunity for more studies to be conducted to understand the differences between students in various disciplines regarding the use of AI tools for learning. 

## 6. Limitations of the Study

This study used a self-report survey to collect data. Hence, a longitudinal study could be adopted to examine the relationships explored in this research. The study’s focus on a specific cultural (Saudi Arabia) and institutional context (university/higher education students) may limit its applicability to diverse educational settings. Cultural nuances and institutional variations could significantly affect students’ acceptance and utilization of ChatGPT. The reliance on self-reported data, particularly in surveys, introduces response bias. Participants may provide socially desirable responses, affecting the potential accuracy of the reported attitudes and behaviors toward ChatGPT. Future studies could adopt a longitudinal approach to explore the temporal dynamics of students’ adoption of AI tools. This would allow for a more nuanced understanding of how these relationships evolve over an extended period. This study has one more limitation, as it did not explore other possible variables that might shape ChatGPT acceptance and usage, such as prior exposure to AI tools, access to technology, and socio-economic status. The inclusion of these controls and variables would enhance our understanding of the evaluated relationships. Therefore, considering such limitations and conducting future studies would contribute to a more comprehensive and nuanced understanding of the moderating roles of gender and study discipline in the acceptance and usage of ChatGPT among university students.

## Figures and Tables

**Table 1:** Students’ profiles (N = 500).

Profile		Freq.	%
Gender	Male	306	61.2
	Female	194	38.8
Age	Less than 20 years	196	39.2
	20 to 25 years	261	52.2
	26 to 30 years	43	8.6
Study discipline	Social sciences	156	31.2
	Humanities	106	21.2
	Natural sciences	111	22.2
	Applied sciences	81	16.2
	Basic sciences	46	9.2

**Table 2:** Factors and psychometric properties.

	Loadings	α	C.R.	AVE	VIF
Performance Expectancy		0.875	0.907	0.712	
PE1	0.732				3.525
PE2	0.740				3.306
PE3	0.936				2.537
PE4	0.943				2.372
Effort Expectancy		0.940	0.956	0.846	
EE1	0.919				3.737
EE2	0.923				4.450
EE3	0.932				3.947
EE4	0.905				3.587
Social Influence		0.842	0.905	0.763	
SI1	0.941				4.183
SI2	0.945				4.285
SI3	0.715				1.419
Facilitating Conditions		0.941	0.953	0.835	
FC1	0.877				4.678
FC2	0.873				4.406
FC3	0.959				3.963
FC4	0.944				4.186
Behaviour Intention (mediating variable)		0.752	0.859	0.672	
M1	0.844				1.879
M2	0.896				2.001
M3	0.708				1.269
Usage (dependent varaiables)		0.928	0.954	0.874	
Y1	0.948				4.130
Y2	0.928				3.325
Y3	0.928				3.734

**Table 3:** Discriminant validity based on the Fornell and Larcker and HTMT methods.

	BI	EE	FC	PE	SI	ChatGPT Usage
BI	**0.820**					
EE	−0.060 [0.095]	**0.920**				
FC	−0.196 [0.195]	0.487 [0.547]	**0.914**			
PE	0.148 [0.195]	0.016 [0.028]	−0.048 [0.061]	**0.844**		
SI	0.546 [0.688]	−0.094 [0.112]	−0.059 [0.059]	−0.076 [0.158]	**0.874**	
ChatGPT Usage	0.800 [0.829]	−0.082 [0.085]	−0.162 [0.142]	0.223 [0.233]	0.486 [0.532]	**0.935**

Note: old values refers to the square root of the AVE for each construct.

**Table 4:** Hypotheses results.

Hypotheses	β	*t*	*p* Values	Results
Performance Expectancy -> Behavioral Intention	0.179	5.083	0.000	Support H1
Effort Expectancy -> Behavioral Intention	0.084	2.085	0.037	Support H2
Social Influence -> Behavioral Intention	0.556	9.570	0.000	Support H3
Facilitating Conditions -> Behavioral Intention	−0.196	4.758	0.000	Reject H4
Performance Expectancy -> Use ChatGPT	0.099	2.213	0.027	Support H5
Effort Expectancy -> Use ChatGPT	−0.051	1.073	0.283	Reject H6
Social Influence -> Use ChatGPT	0.132	2.390	0.017	Support H7
Facilitating Conditions -> Use ChatGPT	0.030	0.534	0.593	Reject H8
Behavioral Intention -> Use ChatGPT	0.743	20.283	0.000	Support H9
Moderation				
Gender x Performance Expectancy -> Use ChatGPT	0.096	1.960	0.050	Support H10
Gender x Effort Expectancy -> Use ChatGPT	0.004	0.076	0.940	Reject H11
Gender x Social Influence -> Use ChatGPT	−0.146	1.962	0.049	Support H12
Gender x Facilitating Conditions -> Use ChatGPT	−0.030	0.538	0.591	Reject H13
Discipline x Performance Expectancy -> Use ChatGPT	−0.034	0.718	0.473	Reject H14
Discipline x Effort Expectancy -> Use ChatGPT	0.021	0.402	0.688	Reject H15
Discipline x Social Influence -> Use ChatGPT	0.093	1.961	0.050	Support H16
Discipline x Facilitating Conditions -> Use ChatGPT	0.007	0.107	0.915	Reject H17

## Data Availability

Data are available upon request from researchers who meet the eligibility criteria. Kindly contact the first author privately through e-mail.

## Appendix A. The Employed Measures

**Factors/Code**	**Full Sentence**
Performance Expectancy	
PE1	“ChatGPT is an invaluable asset for enhancing my academic endeavors”
PE2	“Employing ChatGPT significantly increases the likelihood of achieving crucial objectives in academic pursuits”
PE3	“ ChatGPT optimizes productivity in academic endeavors by streamlining task and project completion”
PE4	“Engaging with ChatGPT has the potential to enhance my academic performance”
Effort Expectancy	
EE1	“I perceive learning to use ChatGPT as straightforward”
EE2	“The interaction with ChatGPT is clear and easily understandable”
EE3	“ChatGPT boasts a user-friendly and intuitive interface”
EE4	“I effortlessly develop proficiency in utilizing ChatGPT”
Social Influence	
SI1	“The individuals who hold significant influence in my life strongly advocate for the use of ChatGPT”
SI2	“The people who influence my actions endorse the utilization of ChatGPT”
SI3	“The perspectives of those whom I deeply respect indicate a recommendation for incorporating ChatGPT into my activities”
Facilitating Conditions	
FC1	“I possess sufficient resources to effectively utilize ChatGPT”
FC2	“I have acquired the necessary skills to proficiently use ChatGPT”
FC3	“ChatGPT aligns with the technological tools I employ”
FC4	“In the event of challenges with ChatGPT, external support and assistance are readily available”
Behaviour Intention [mediating variable]	
M1	“I have made the decision to persist in using ChatGPT going forward”
M2	“I am fully committed to employing ChatGPT as an integral tool for my academic endeavors”
M3	“I am determined to maintain a consistent usage of ChatGPT”
Usage [dependent varaiable]	
Y1	“I plan to apply the knowledge and skills gained from ChatGPT in my educational pursuits”
Y2	“The knowledge and skills acquired through ChatGPT will prove beneficial to my classroom endeavors”
Y3	“Utilizing ChatGPT has contributed to enhancing my academic performance”
